# Enhanced CDC of B cell chronic lymphocytic leukemia cells mediated by rituximab combined with a novel anti-complement factor H antibody

**DOI:** 10.1371/journal.pone.0179841

**Published:** 2017-06-28

**Authors:** Mark T. Winkler, Ryan T. Bushey, Elizabeth B. Gottlin, Michael J. Campa, Eross S. Guadalupe, Alicia D. Volkheimer, J. Brice Weinberg, Edward F. Patz

**Affiliations:** 1 Department of Radiology, Duke University Medical Center, Durham, North Carolina, United States of America; 2 Department of Medicine, Duke University Medical Center, Durham, North Carolina, United States of America; 3 Durham VA Medical Center, Durham, North Carolina, United States of America; 4 Department of Immunology, Duke University Medical Center, Durham, North Carolina, United States of America; 5 Department of Pharmacology & Cancer Biology, Duke University Medical Center, Durham, North Carolina, United States of America; University of Texas MD Anderson Cancer Center, UNITED STATES

## Abstract

Rituximab therapy for B cell chronic lymphocytic leukemia (B-CLL) has met with mixed success. Among several factors to which resistance can be attributed is failure to activate complement dependent cytotoxicity (CDC) due to protective complement regulatory proteins, including the soluble regulator complement factor H (CFH). We hypothesized that rituximab killing of non-responsive B-CLL cells could be augmented by a novel human monoclonal antibody against CFH. The B cells from 11 patients with B-CLL were tested *ex vivo* in CDC assays with combinations of CFH monoclonal antibody, rituximab, and a negative control antibody. CDC of rituximab non-responsive malignant B cells from CLL patients could in some cases be augmented by the CFH monoclonal antibody. Antibody-mediated cytotoxicity of cells was dependent upon functional complement. In one case where B-CLL cells were refractory to CDC by the combination of rituximab plus CFH monoclonal antibody, additionally neutralizing the membrane complement regulatory protein CD59 allowed CDC to occur. Inhibiting CDC regulatory proteins such as CFH holds promise for overcoming resistance to rituximab therapy in B-CLL.

## Introduction

The monoclonal antibody (mAb) rituximab targets CD20 and is used alone or in combination with chemotherapy to treat patients with chronic B cell lymphocytic leukemia (B-CLL). However, many patients fail to respond for a variety of reasons. Rituximab, a chimeric mAb composed of human IgG1 constant regions and murine variable regions, is thought to work by three mechanisms: *(i)* direct signaling and activation of internal apoptotic pathways, *(ii)* induction of antibody-dependent cell-mediated cytotoxicity (ADCC), and *(iii)* induction of complement-dependent cytotoxicity (CDC) [1–3].

Several studies suggest that CDC of B-CLL cells by rituximab is ineffective because of the presence of complement regulatory proteins, including the soluble protective protein complement factor H (CFH), that inhibit CDC [4–6]. CFH is responsible for inhibiting alternative pathway CDC, and blocking its activity has been suggested as a strategy to induce CDC in rituximab-resistant patients [7, 8]. CFH binds to host cells via non-covalent interactions with membrane polyanions and C3b and its fragments [9, 10]. C3b, an initiator of the complement cascade, is covalently bound to cell membranes. By several mechanisms, CFH prevents the formation and deposition of additional C3b and propagation of the complement cascade. Inhibiting or inactivating CFH permits activation of complement leading to cell lysis and death.

CFH is a 155 kDa multifunctional, multidomain protein composed of 20 repeating subunits called short consensus repeat (SCR) or complement control protein (CCP) modules [11]. We previously reported the isolation and characterization of a novel human derived monoclonal antibody (mAb) to CFH, which recognizes a conformationally distinct epitope in the SCR19 domain [12]. This region of SCR19 interacts with C3b as deduced from the co-crystal structure of CFH and C3b [13]. We demonstrated that the cloned human CFH mAb promoted CDC in numerous cancer cell lines and inhibited the growth of tumors in mice [12]. The goal of the current study was to investigate the ability of this CFH mAb to promote CDC of malignant B-CLL cells that were resistant to rituximab-mediated CDC *ex vivo*.

## Methods

### Patients

This study was approved by the Institutional Review Boards of our institutions and was HIPAA compliant. All patients were informed of benefits and risks of the research, and all gave written consent for the research to proceed. Patients were followed longitudinally in our clinics. We obtained peripheral venous blood samples on return clinic visits. For this study, blood samples were acquired from 11 B-CLL patients as patients were seen in the hematology-oncology clinics at the Duke University and Durham VA Medical Centers. None of these patients had received any treatment before the blood was collected. Table 1 displays detailed characteristics of the patients and their B-CLL cells. B cells were isolated from peripheral blood using a B cell enrichment cocktail (RosetteSep-B Cell, Stemcell Technologies, Vancouver, BC) [14]. The processed cells were greater than 90% B-CLL cells. Cells were characterized for CD38 and Zap70 expression by flow cytometry, immunoglobulin gene heavy chain (IGVH) mutation status by DNA sequencing, and cytogenetics abnormalities for 13q deletion, trisomy 12, 11q deletion, and 17p deletion by fluorescent in situ hybridization (FISH) as previously described [14]. Rai staging was done according to Hallek and others [15]. The median follow-up time was 5 years. None of the patients died. Serum samples from each patient were also acquired to evaluate baseline cytotoxicity and complement activity prior to therapy.

**Table 1:** Patient demographics, B-CLL status, and B cell characterization.

Patient demographics and B-CLL status					B cell characterization			
Patient number	Age at diagnosis	Gender	Race	Rai stage	CD38	Zap70	IGVH	Cyto-genetics
1	63	M	Black	1	Pos	Pos	Unmut	Tri12
2	52	M	White	0	Neg	Neg	Mutated	13q del
3	75	M	White	0	Neg	Pos	Unknown	Normal
4	60	F	White	0	Neg	Pos	Mutated	13q del
5	84	M	White	0	Neg	Pos	Unmut	11q del
6	67	F	White	0	Neg	Neg	Mutated	13q del
7	71	F	White	0	Neg	Pos	Mutated	13q del
8	58	M	White	0	Neg	Pos	Unmut	13q del
9	61	M	Black	1	Neg	Neg	Unmut	11q del
10	63	F	White	1	Pos	Pos	Unmut	11q del
11	61	M	Black	2	Pos	Neg	Unmut	13q del

The length of time to initiation of treatment from the date of diagnosis was defined as the time-to-treatment [16]. The median follow-up time was 5 years. None of the patients died. Partial remission, complete remission, and no response were determined as described before [15].

### Monoclonal antibodies

Rituximab (Genentech, San Francisco, CA) was acquired through a drug exchange program at Duke University Medical Center, Department of Hematology and Oncology. Human CFH mAb 7968 was generated by cloning and expressing antibody genes derived from a single B cell as previously described [12]. The IgG1subclass-matched negative control antibody 7B2 recognizes gp41, an HIV-1 envelope glycoprotein, and was a gift of Dr. H. Liao of the Duke Human Vaccine Institute. The rat anti-human CD59 mAb YTH53.1 was obtained from Santa Cruz Biotechnology (Dallas, TX). The FITC mouse anti-human CD20 mAb 2H7 was obtained from BD Biosciences (San Jose, CA).

### Cytotoxicity assay

A lactate dehydrogenase (LDH) release assay (CytoTox96, Promega, Durham, NC) was used to assess CDC of patient B cells in the presence of rituximab, with or without the CFH mAb. Normal human serum (NHS; Complement Technology, Inc., Tyler, TX) with known complement activity or, alternatively, each patient’s own serum (PS), was used as the source of complement in this assay. Standard controls included heat-inactivated NHS and PS (both treated for 1 h at 56°C), and when applicable, the negative control IgG1 subclass-matched mAb 7B2.

Cytotoxicity assays were performed as follows: Purified B-CLL cells were placed in 96-well tissue culture plates at 5 x 10^5^ cells per well. Serum and antibodies were then mixed with cells in RPMI-1640 culture medium: Serum was added at a 1:16 dilution, and rituximab, the CFH mAb, or control mAb was added at 100 μg/ml, 200 μg/ml, and 200 μg/ml, respectively (final concentrations). In some experiments, the CD59 mAb was added at 100 μg/ml. After addition of the serum and antibodies to B-CLL cells in medium, the assay plate was incubated at 37°C for 2 h. The plate was then centrifuged at 250 x g, and the supernatants were removed to an assay plate and treated to generate red formazan product that is proportional to the number of lysed cells. The assay plate was read with a plate reader at 490 nm (SpectroStar Nano BMG, LabTech, Ortenberg, Germany) and percent cytotoxicity calculated. Each experimental condition was performed in triplicate and mean cytotoxicities were obtained, along with standard deviations. Data were normalized as described in the figure legends. P values were calculated for relevant comparisons using Student’s t-test.

Patients whose B-CLL cells were able to be lysed when rituximab was added (p< 0.05 vs. no rituximab) were termed “responders.” Patients whose B-CLL cells were able to be lysed to a significantly greater extent when the CFH mAb was added to rituximab (p< 0.05 vs. control mAb and rituximab) in the presence of either NHS or their own serum were termed “augmentable responders.”

### Flow cytometry analysis of CD20 and CD59

Patient B-CLL cells were analyzed via flow cytometry for relative expression levels of CD20 and CD59 using methods described previously [17]. The membrane complement regulatory protein (mCRP) CD59 was previously shown to be present on B-CLL cells. [18]. For each analysis, 5 x 10^5^ cells were incubated in duplicate with 20 μl of a primary FITC labeled CD20 antibody at 50 μg/ml, or 5 μl of a rat primary antibody against CD59 (YTH53.1) at 200 μg/ml followed by 5 μl of FITC (200 μg/ml) conjugated secondary labeled antibody against rat IgG (Jackson ImmunoResearch, West Grove, PA). Flow cytometry was performed using a FACSCanto II (BD Biosciences, San Jose, CA). Since measurements of the two markers were conducted on subsets of 10 B-CLL samples on three different days, fluorescence intensity (FI) data for each marker were standardized as follows: The data in Experiments 2 and 3 were standardized to those in Experiment 1 by multiplying FI for each data point by the ratio (FI for patient #6 in Experiment 1/FI for patient #6 in the current experiment).

### Complement activity assay

Because previous studies have suggested that there are complement deficiencies in B-CLL patients that may limit rituximab efficacy, [19] the complement activity in each patient’s serum was assessed using a standard hemolytic assay measuring the complement-mediated lysis of sheep red blood cells (RBC) coated with rabbit anti-sheep erythrocyte antiserum (hemolysin), as described by Complement Technology, Inc. (Tyler, TX), from whom cells and reagents for this assay were obtained (http://www.complementtech.com/product-description/Cells/EA.htm).

## Results

### Baseline rituximab cytotoxicity of B-CLL cells from 11 patients

**Fig 1:**
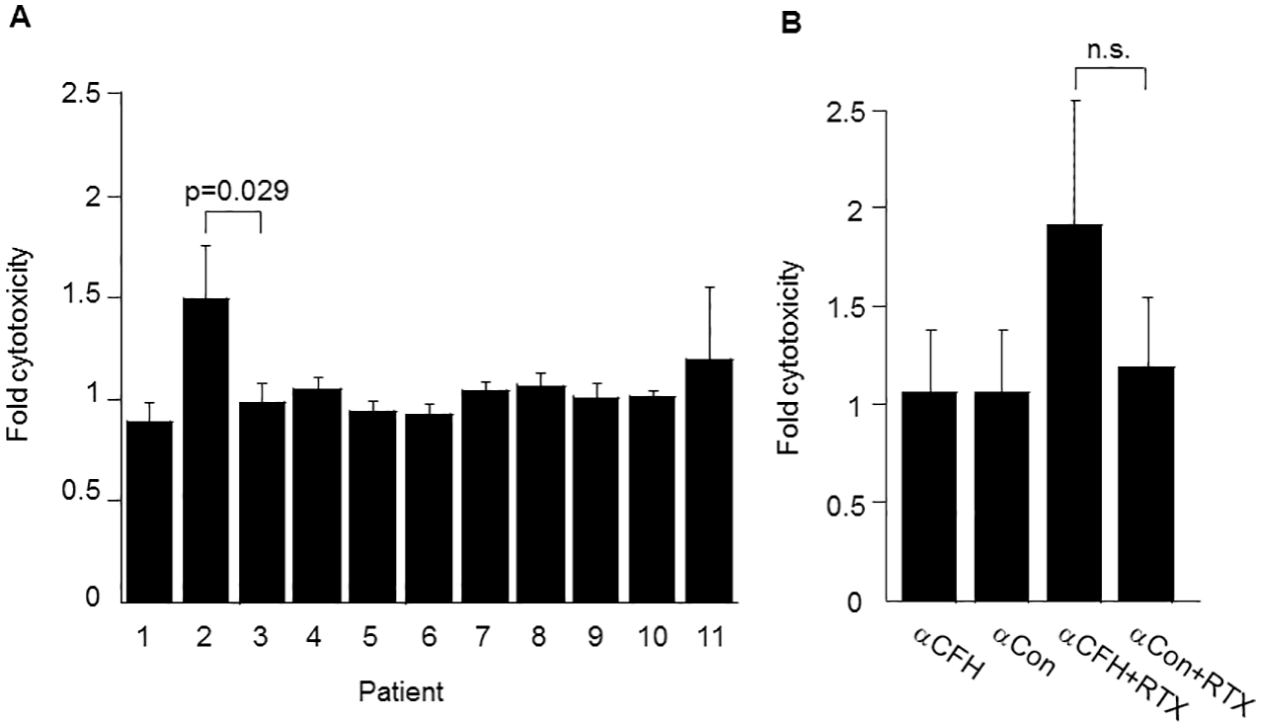
CDC of B-CLL cells. (A) CDC of B-CLL cells in the presence and absence of rituximab (RTX). B-CLL cells from each of 11 CLL patients were treated with RTX or left untreated, with NHS added as a source of complement. Mean percent CDC in the presence of RTX was divided by the mean percent CDC in the absence of RTX to obtain "fold cytotoxicity" for each patient. (B) CDC of B-CLL cells from patient 11 treated with a CFH mAb or control mAb in the presence or absence of RTX. B-CLL cells from this patient were treated with CFH mAb7968 or isotype-matched negative control antibody 7B2 with or without RTX, with NHS added as a source of complement. Mean percent CDC was divided by the mean percent CDC of the “no antibody” control to obtain "fold cytotoxicity" for each condition.

The B cells from 11 B-CLL patients were tested in CDC assays to determine whether or not they were rituximab sensitive (Fig 1A). In these assays, cells were incubated with NHS as a source of complement either with or without rituximab, and CDC was measured by LDH release. As part of a second experiment using the cells of patient #11, a negative control antibody was added with rituximab and compared to the negative control antibody alone to determine baseline cytotoxicity (Fig 1B). Ten of the 11 patients (age 65 ± 9 years [SD]) were classified as non-responders as their B cells had no statistically significant increase in cytotoxicity with rituximab over the negative (no antibody) control (p>0.05); one patient (patient #2, a 52 year old male) was classified as a responder to rituximab, as this patient’s B cells demonstrated a 1.5 fold increase in cytotoxicity with rituximab over the negative control (p = 0.014). The response of the B-CLL cells of patient #2 to rituximab was also statistically significant as compared to the B-CLL cells of patient #3 that showed no response (p = 0.029). (Fig 1A). Clinically, patient #2 was eventually treated with ibrutinib and rituximab and had a clinical partial response with a decrease in lymph node size after treatment.

Overall, the patients were early stage (7 Rai stage 0, 3 stage 1, and 1 stage 2), but several had poor prognostic markers (6 of 11 had unmutated immunoglobulin heavy chain gene [IGVH], and 7 of 11 had Zap-70 positive B-CLL cells) (Table 1). None of the 11 had been treated before our *ex vivo* testing. However, 3 of the 11 (patients 2, 8, and 10) were eventually treated with rituximab in combination with other drugs (1 with bendamustine and 2 with ibrutinib). All three of the patients receiving rituximab and other drugs had clinical responses with reduced leukemia cell counts and decreased lymph node sizes (Table 2), but it was not possible to ascertain if the responses were due to the rituximab or the other drugs.

**Table 2:** Patient treatment history.

Patient number	Date entered study	Treated?	TTT (yrs)	Received RTX	Drug regimen 1	Drug regimen 2
1	7-13-11	No	4.5	NA	NA	NA
2	10-15-07	Yes	17.4	Yes	2015; IR (PR)	NA
3	8-8-13	No	3.1	NA	NA	NA
4	10-7-09	No	7.3	NA	NA	NA
5	6-9-14	Yes	1.9	No	2015; Ob/CMB (CR)	NA
6	12-15-10	Yes	6.6	No	2016; I (PR)	NA
7	1-5-11	No	5.2	NA	NA	NA
8	11-28-07	Yes	6.0	Yes	2006; CMB (NR)	2015; BR (CR)
9	3-4-15	No	1.5	NA	NA	NA
10	3-3-10	Yes	5.5	Yes	2015; IR (PR)	NA
11	1-5-15	Yes	4.1	Yes	2015; BR (PR)	2015; I (PR)

Abbreviations are: TTT, time-to-treatment (TTT), PR, Partial remission, CR, complete remission, NR, no response, IR, ibrutinib + rituximab; Ob/CMB, obinutuzumab + chlorambucil; I, ibrutinib; FCR, fludarabine + cyclophosphamide + rituximab; BR, bendamustine + rituximab. NA signifies not applicable.

### CFH mAb promotes CDC of rituximab-refractory B-CLL cells

**Fig 2:**
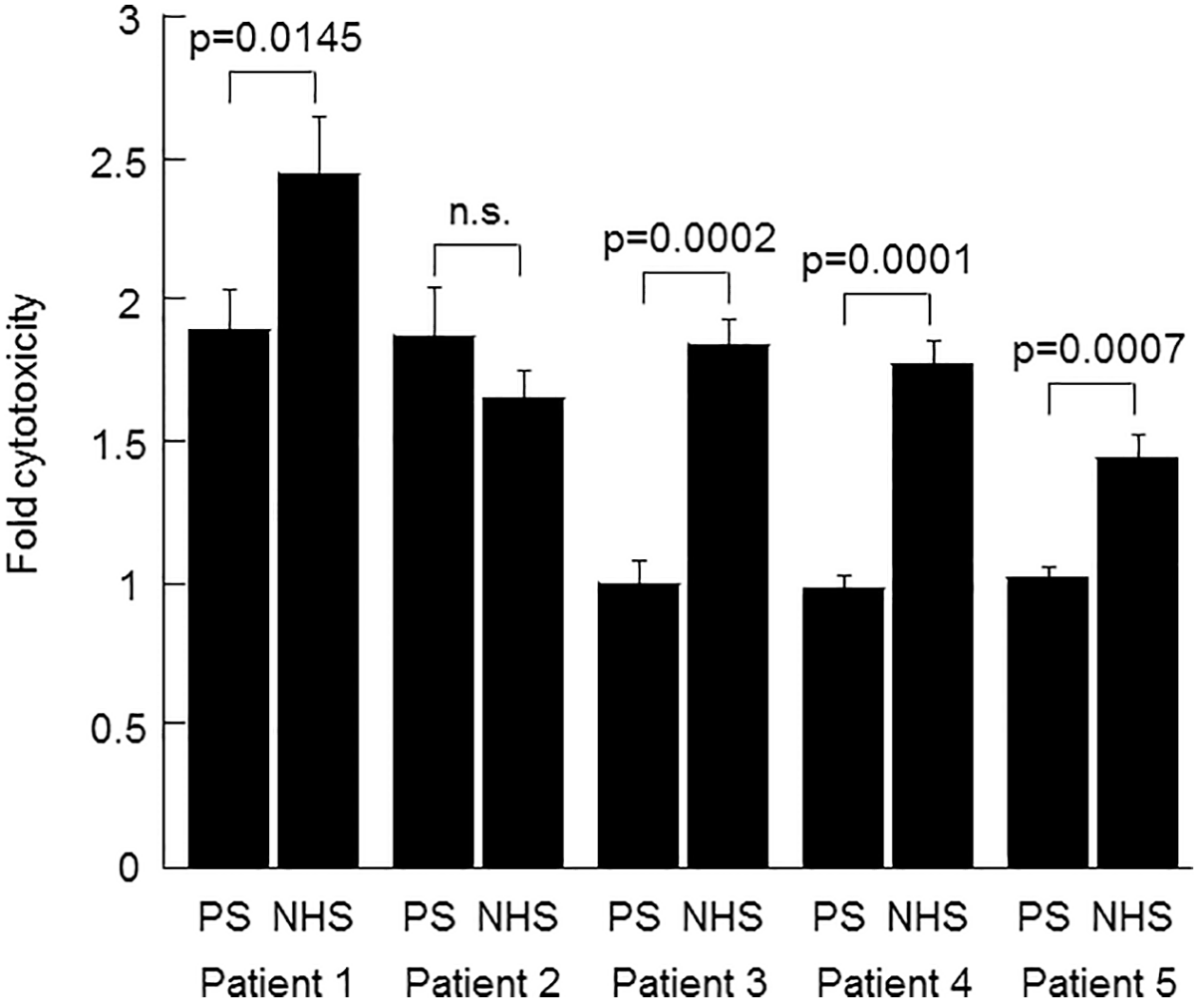
Antibody-induced CDC of B-CLL cells from five CLL patients in the presence of PS or NHS. Panels show B-CLL cell cytotoxicity with the autologous patient serum (PS) from patients 1–5, respectively, in comparison with cytotoxicity using NHS. Cytotoxicity was induced in the presence of RTX plus the addition of either CFH mAb7968 (α-CFH) or isotype-matched negative control antibody 7B2 (α-Con). For each patient and serum type, the mean percent CDC in the presence of the αCFH antibody was divided by the mean percent CDC in the presence of the αCon antibody to obtain "fold cytotoxicity". The p values of the difference between response in PS vs. NHS are shown on the figure. The p values for fold cytotoxicity as a result of α-CFH addition compared to α-Con addition were 0.00017, 0.00060, n.s., n.s., n.s in PS, and 0.00016, 0.00021, 0.000012, 0.00001, and 0.00025 in NHS, for patients 1–5 respectively (n.s = not significant).

**Fig 3:**
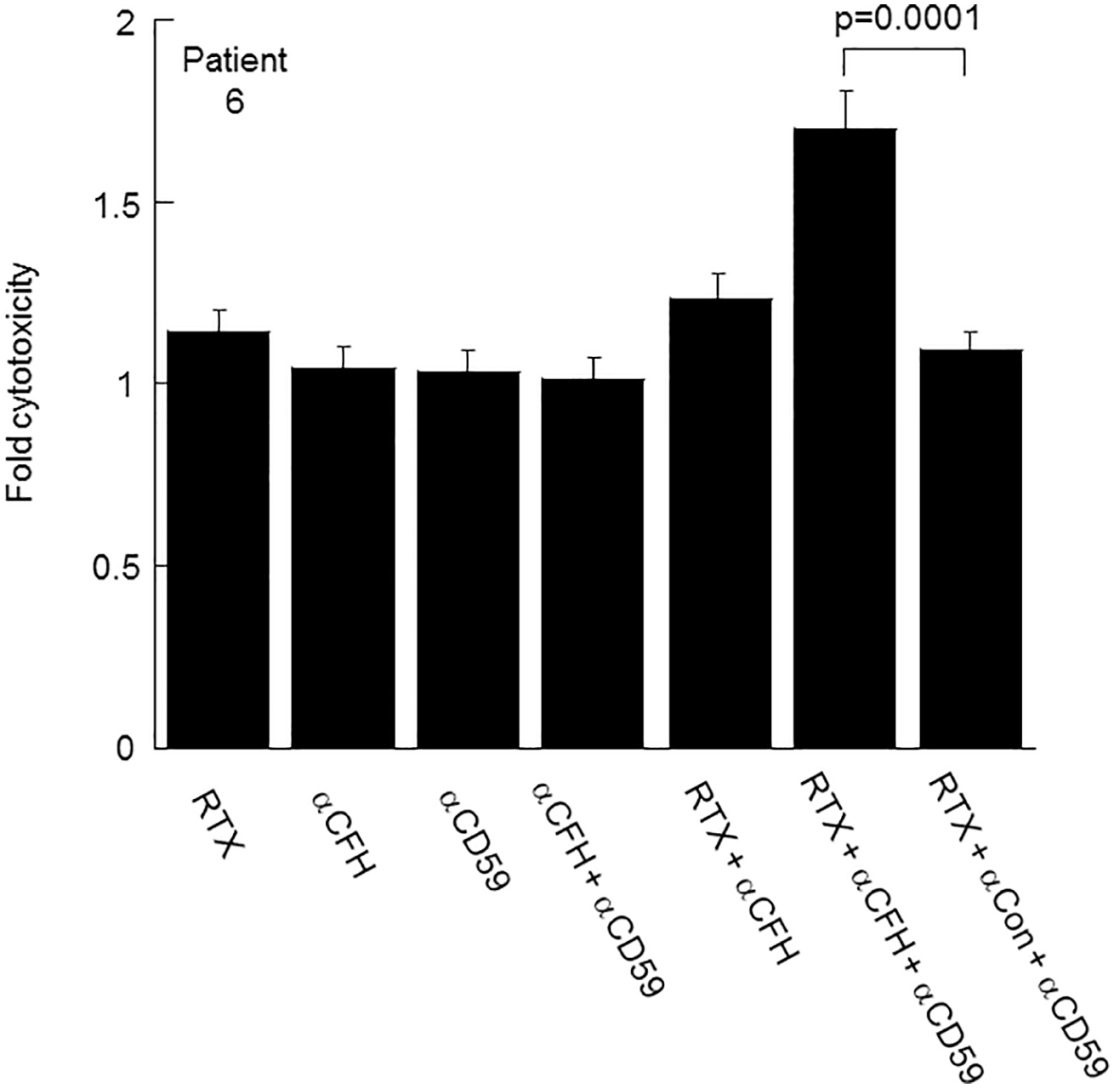
The effect of anti-CD59 antibody on CDC of RTX/CFH MAb nonresponder B-CLL cells with high mean CD59 expression levels (Patient #6). CDC reactions were carried out with NHS as a complement source. For each condition, the mean percent CDC was divided by the mean percent CDC of the αCon antibody to obtain "fold cytotoxicity". The CDC assay for the α-CFH + RTX condition was performed on a different day from the other conditions.

To test the effect of the CFH mAb on tumor cell lysis, B cells isolated from the peripheral blood in the 11 CLL patients were incubated with serum as a source of complement, plus a combination of antibodies (rituximab, CFH mAb7968, or IgG1 subclass-matched negative control mAb7B2) in CDC assays. In the first patient tested (#11), neither the CFH mAb alone nor rituximab plus a negative control mAb resulted in measurable CDC over background. However, when rituximab and the CFH mAb were added together, CDC of the B-CLL cells of this patient increased nearly two-fold over background (Fig 1B) although this effect failed to reach statistical significance (p = 0.16). In 5 of 11 patients there was a significant increase (p<0.05) in CDC with the addition of the CFH mAb7968 to rituximab vs. control mAb plus rituximab (Fig 2). Thus, 45% of patients were considered augmentable responders. These included the one rituximab responder (patient #2), and five of the rituximab non-responders, (patient # 1, 3, 4, and 5). The B-CLL cells of 6 of the 11 patients (patient # 6, 7, 8, 9, 10 and 11) did not respond to a combination of rituximab plus CFH mAb7968 regardless of serum type (data shown for patient 6 in NHS [Fig 3]). In all cases where enhanced cytotoxicity was observed, heat-inactivation of the serum abolished it, proving complement dependence.

CDC of the B-CLL cells of all 5 augmentable responders was enhanced by the addition of the αCFH antibody to RTX in NHS by factors of 1.5–2.5. The situation was different when using autologous patient sera: CDC of the B-CLL cells of patients 1 and 2 was enhanced by the addition of the αCFH antibody to RTX in PS whereas the B-CLL cells of patients 3, 4, and 5 were not (Fig 2).

### Complement activity in the serum

**Fig 4:**
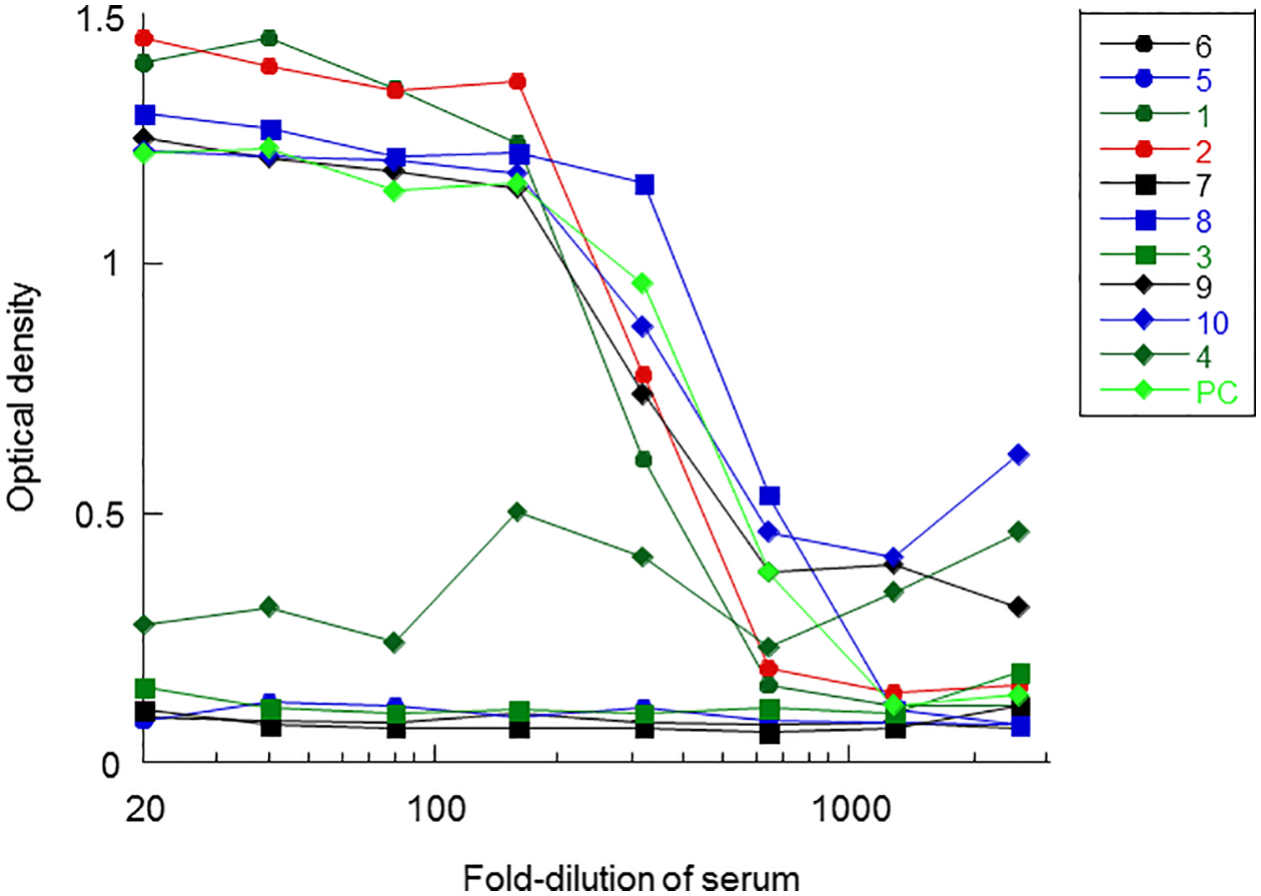
Sheep RBC hemolysis assay for complement activity. B-CLL patient sera and a positive control (PC) serum (light green diamonds) were diluted and assayed at each dilution for the ability to lyse sheep RBC.

Complement activity was assayed in the serum of 10 of the 11 patients. The three augmentable responder patients whose B-CLL cells failed to respond to the αCFH antibody in the presence of their own serum (patients 3, 4, and 5) had low levels of complement activity, whereas two augmentable responders whose B-CLL cells did respond to the αCFH antibody in the presence of their own serum (patients 1 and 2) had normal levels of complement activity (Fig 4). In all, 5 of the 10 B-CLL patients (50%) had low serum complement activity (patients 3, 4, 5, 6, and 7).

### Expression of CD20 and CD59 and the effect of an anti-CD59 antibody on CDC

The B cells from 10 of the 11 B-CLL patients were further analyzed by flow cytometry to determine expression levels of CD20 and the complement inhibitor protein CD59. To classify relative membrane expression level of these two proteins, the range of individual mean FITC signals for each protein was determined for all patients and then roughly divided into thirds. Each patient was then assigned a relative expression level based on whether their respective mean FITC signal fell within the lowest 1/3 (signifying relatively low expression), middle 1/3 (signifying moderate expression), or highest 1/3 (signifying high expression) for the aggregate 10 samples (Table 3**)**.

**Table 3:** B cell CDC response to mAbs, serum complement activity, CD20 level, and CD59 level for B-CLL patients.

	Classification of B-CLL cells by CDC response^a^ in the presence of:			Complement activity^b^	Relative CD20 level^c^	Relative CD59 level^c^
Patient number	NHS+RTX	NHS+RTX+CFH mAb	PS+RTX+CFH mAb			
1	NR	R	R	high	mod	mod
2	R	R	R	high	mod	low
3	NR	R	NR	low	high	high
4	NR	R	NR	low	high	mod
5	NR	R	NR	low	mod	low
6	NR	NR	NR	low	mod	high
7	NR	NR	NR	low	high	mod
8	NR	NR	NR	high	low	high
9	NR	NR	NR	high	low	low
10	NR	NR	NR	high	low	high
11	n.t ^d^	R	n.t.	n.t.	n.t.	n.t.

^a^R = responder; NR = non-responder in CDC assay based on a significant difference (p<0.05) between test and control condition. As defined in the text, an “augmentable responder” is a patient whose B-CLL cells can be lysed in the presence of *both* antibodies together in *either* NHS or PS. By this definition, #1,2,3,4, and 5 are augmentable responders.

^b^low = non-functional complement; high = functional complement in hemolytic assay

^c^low, mod, high = low, moderate, or high expression within cohort, divided in thirds

^d^n.t. = not tested

Three of the non-responders to the rituximab plus CFH mAb combination (patients #6, 8, and 10) had a relatively low to moderate level of CD20 and a relatively high level of CD59, suggesting that these patients’ B-CLL cells had at least two potential mechanisms of resistance. Given the importance of CD59 in preventing the formation of the membrane attack complex at the cellular surface, we asked if CD59 could be a factor restricting CDC of B-CLL cells of a patient who did not respond to the combination of the two antibodies. The B-CLL cells of patient #6 were not lysed with rituximab plus the CFH mAb, but the addition of a CD59 mAb resulted in a significant increase in CDC (Fig 3). Like patient #6, the B-CLL cells of patient #8 were non-responsive to rituximab plus the CFH mAb, but the addition of an anti-CD59 mAb to the B-CLL cells of patient #8 did not increase CDC (data not shown). We also tested the B-CLL cells of patient #3, an augmentable responder with both high CD59 and high CD20, whose B-CLL cells were lysed in the presence of both rituximab and the CFH mAb. The CD59 mAb did not increase CDC of these B-CLL cells further (data not shown).

## Discussion

Despite recent advances in the treatment of chronic B-CLL, this leukemia remains essentially incurable [20, 21]. The development of Bruton tyrosine kinase (ibrutinib), phosphoinositide 3-kinase (idelalisib), and B-cell lymphoma 2 (bcl-2) (venetoclax) inhibitors, as well as novel anti-CD20 antibodies (obinutuzumab and ofatumumab), have provided new options for therapy [20]. Guidelines for therapy vary, but most divide patients based on their general "fitness," and presence of the p53 gene (17p) deletion or p53 mutation [20, 21]. For fit individuals without 17p deletion, most recommend combination therapy with fludarabine, cyclophosphamide, and rituximab (FCR) if < 65 years old, or bendamustine and rituximab if > 65 years old. If 17p deletion is present and the patient is fit, they are given ibrutinib alone (or idelalisib with rituximab if unable to receive ibrutinib) or considered for a stem cell transplant. If 17p deletion is present and the patient is considered unfit, they are generally treated the same but considered for a drug clinical trial (and not a transplant) [20, 21]. Other options after failure include treatment with the bcl-2 inhibitor venetoclax. Thus, rituximab has played a central role in the treatment of CLL.

Although rituximab has been a widely used mAb, many patients are resistant to its effects as a monotherapy. At least one of the resistance mechanisms is thought to be due to the presence of complement regulatory proteins, preventing the antibody from causing CDC. Hörl et al. showed that inhibiting CFH binding to host cell surfaces, through the use of recombinant human CFH-derived SCR18-20, sensitizes rituximab non-responding B cells to CDC with therapeutic mAbs [4, 22]. However, use of this peptide therapeutically is not a viable strategy since high concentrations of soluble plasma CFH would compete for its binding to cells. In the current study, we used a fully human-derived anti-CFH mAb that recognizes a cryptic epitope in SCR19 that is essential for CFH function. This antibody, with the same specificity as the autoantibodies noted in some patients with non-metastatic NSCLC, kills tumor cells but does not recognize soluble plasma CFH [12]. NSCLC patients with these CFH autoantibodies show no symptoms indicative of an off-target effect attributable to its presence [23]. Here we demonstrate that the CFH mAb sensitizes rituximab non-responsive B cells to CDC with rituximab and suggest that the CFH mAb may augment rituximab therapy in some cases of rituximab-refractory CLL.

Failure of rituximab therapy has been attributed to low CD20 expression [8] or failure of one of the effector mechanisms that include induction of antibody-dependent cell-mediated cytotoxicity (ADCC) and induction of complement dependent cytotoxicity (CDC) [1–3]. Deficiencies in serum complement proteins are also thought to contribute in some cases to limiting CD20-directed therapy [19]. Furthermore, tumor cells possess mechanisms to protect themselves from CDC using both mCRPs and soluble CFH. Blocking CD59, one of the mCRPs, strongly enhanced the cytotoxic effects of rituximab against lymphoma cell lines [24]. Downregulation of the expression of mCRPs CD46, CD55, and CD59 improved the efficacy of rituximab and the anti-CD20 mAb ofatumumab in lymphoma and leukemia cell lines and primary CLL samples [6, 25].

In our cohort of patients, there were two (#3 and #4) whose B-CLL cells had relatively high levels of CD20 yet were non-responsive to rituximab *in vitro*. These malignant B cells were responsive to a combination of rituximab and the CFH mAb. There were also 4 patients with relatively high expression of CD59 (#3, 6, 8, and 10). One of these (#6), whose B-CLL cells were resistant to the combination of rituximab and the CFH mAb, had a significant increase in CDC with the addition of a CD59 mAb. However, the B-CLL cells of two other patients with high CD59 (#3 and #8) could not be lysed by the addition of CD59 mAb. Cells from patient #10 were not tested. These data suggest that tumor cells use multiple different strategies to protect against CDC, and that understanding each of these potential inhibitory mechanisms will be required to achieve a relevant therapeutic response.

The importance of complement activity in patients receiving rituximab therapy has been established previously [19]. The authors hypothesized that patients with CLL receiving anti-CD20 antibody therapy may have exhausted complement from repeated rounds of rituximab-induced CDC and, therefore, could benefit from supplementation with fresh frozen plasma (FFP) as a source of complement concurrently with rituximab therapy [19]. In total, 5 of 10 patients in our study had low complement activity levels, and this correlated with a lack of response to the double antibody combination when a patient’s own serum was used. However, no patients in this B-CLL cohort had received rituximab or any other anti-CD20 antibody therapies before our *ex vivo* testing. This suggests that testing complement activity in B-CLL patients should be considered prior to rituximab therapy.

Combining rituximab with a novel CFH mAb may be a new way to improve treatment of B-CLL. Combination therapy is becoming an increasingly common approach for treating a variety of neoplasms. Our results suggest that CDC of malignant B-CLL cells can be augmented when rituximab is used with a CFH mAb specific to tumor cells, even when the cells are rituximab non-responsive. For patients whose B-CLL cells do not show increased CDC with the rituximab-CFH mAb combination, the addition of antibodies against mCRPs including CD59 might lead to induction of CDC and increased cellular cytotoxicity. Lastly, the administration of FFP may be necessary to restore the complement cascade in patients receiving mAb therapies that require complement for action, or in patients who have low serum complement activity for other reasons. Further studies will be necessary to determine the safety and efficacy of the CFH mAb in treating B-CLL as well as other neoplasms for which rituximab is used. It is becoming increasingly clear that tumors use multiple mechanisms to evade the immune system and are often resistant to monotherapy. A better understanding of resistance mechanisms will help optimize cancer therapy.

## Supporting information

S1 File Original data underlying means, SDs, and p values for CDC and flow cytometry assays. Table A. Responsiveness of B-CLL cells to RTX. Table B. Responsiveness of the B-CLL cells of patient 11 to αCFH vs. αCFH + RTX. Table C. Response of B-CLL cells to the αCFH antibody in the presence of patient serum vs. NHS. Table D. CDC of B-CLL cells in the presence of different combinations of antibodies. Table E. Flow cytometry original data in support of Table 3.
(XLSX)
